# Retrospective Comparison of Stiff Wire-Based 2D3D, Traditional 3D3D Image Fusion, and Non-Image Fusion Techniques and Their Role in Thoracic Aortic Endovascular Repair

**DOI:** 10.3390/jcm14020301

**Published:** 2025-01-07

**Authors:** Peter Osztrogonacz, Zsolt Garami, Alan B. Lumsden, Csaba Csobay-Novák, Ponraj Chinnadurai

**Affiliations:** 1 Department of Cardiovascular Surgery, Houston Methodist Hospital, 6550 Fannin St, Houston, TX 77030, USA; zgarami@houstonmethodist.org (Z.G.); ablumsden@houstonmethodist.org (A.B.L.); pchinnadurai@houstonmethodist.org (P.C.); 2 Department of Vascular and Endovascular Surgery, Semmelweis University, 68 Városmajor St, 1122 Budapest, Hungary; 3 Department of Interventional Radiology, Semmelweis University, 68 Városmajor St, 1122 Budapest, Hungary; csobay.csaba@semmelweis.hu; 4 Occam Labs, 3.6 Bank Studios Park, Royal Road, London NW10 7LQ, UK

**Keywords:** thoracic endovascular aortic repair, TEVAR, descending thoracic aorta, fiducial markers, image guidance

## Abstract

**Objective**: The aim of this study was to compare the outcomes of stiff wire-based 2D3D, 3D3D image fusion (IF), and non-image fusion techniques for simple zone 2 and zone 3 TEVAR cases in terms of radiation exposure, contrast dose, and fusion and projection accuracy. **Methods**: A single-center retrospective observational study was conducted based on data gathered from patients who underwent TEVAR between 2016 and 2023 at our tertiary aortic referral center. Those who underwent Z2 and Z3 TEVAR during the indicated period were included. The dose area product and number of DSAs were considered as primary outcomes, while projection accuracy and image fusion accuracy were considered as secondary outcomes. **Results**: A total of 79 patient were included. They were allocated to non-image fusion (NIF, n = 40), 2D3D IF (n = 14), and 3D3D IF (n = 25) groups. DAP was significantly lower both in the NIF [1542.75 µGym^2^ (751.72–3351.25 µGym^2^), *p* = 0.011] and 2D3D IF [1320.1 µGym^2^ (858.57–2572.07 µGym^2^), *p* = 0.013 groups compared to the 3D3D [2758.61 µGym^2^ (2074.73–4772.9 µGym^2^)] cohort. In the Z3 subgroup, DAP was significantly lower in the 2D3D IF group compared to the 3D3D IF group [(1270.84 µGym^2^ (860.56–2144.69 µGym^2^) vs. 2735.76 µGym^2^ (1583.86–5077.23 µGym^2^), *p* = 0.044]. 2D3D image fusion was associated with a significantly lower number of pre-deployment angiographies compared to NIF [1 (1–1) vs. 2 (1–3), *p* = 0.031], which we used as a surrogate for contrast dose. **Conclusions**: The entire study population analysis showed a significantly lower DAP with 2D3D IF compared to 3D3D IF, while there was no significant difference compared to NIF. It seems that stiff wire-based 2D3D IF does not cost in terms of DAP compared to NIF, while it is more favorable compared to 3D3D IF. Additionally, simple Z3 TEVAR cases might be improved by implementing the stiff wire-based 2D3D technique as a result of decreased DAP compared to 3D3D IF and decreased contrast dose compared to NIF.

## 1. Introduction

Over the past two decades, thoracic endovascular aortic repair (TEVAR) has been adopted as an alternative to treat thoracic aortic pathologies due to its more favorable perioperative outcomes [1,2,3]. According to current society guidelines, TEVAR is the primary surgical treatment option for most descending aortic pathologies over open surgical repair, including descending thoracic aortic aneurysms and complicated type B aortic dissection [4,5].

Beside the additional benefits offered by TEVAR in terms of improved perioperative morbidity, mortality, and shorter hospitalization over open surgical repair, it is not without perioperative risks or even long-term detrimental health effects. Perioperative radiation and the use of iodinated contrast agents render patients undergoing TEVAR more susceptible to renal impairment [6,7,8,9,10] and malignant transformation [11,12,13].

There have been efforts in many areas of endovascular surgery to mitigate these risks. For example, 2D3D and 3D3D fusion-based image guidance offer reduced contrast dose and radiation exposure during EVAR [14,15,16,17,18]. While lowering the radiation burden is of paramount importance from a patient standpoint, long-term exposure could affect medical staff as well. Consequently, efforts to reduce radiation exposure are particularly important not only for patients, but for medical staff managing cases in a radiation environment as well.

From a patient care perspective, these innovations are especially vital in high-risk populations, including those with chronic kidney disease or prior malignancy, who are disproportionately vulnerable to the adverse effects of radiation and contrast agent. For healthcare providers, the cumulative occupational exposure to radiation remains a significant concern, underscoring the necessity of implementing robust protective measures such as lead shielding, dosimetry monitoring, and adherence to the as low as reasonably achievable (ALARA) principle.

The development of novel imaging techniques is critical in advancing the safety and efficacy of TEVAR. Fusion imaging systems allow for the precise overlay of preoperative imaging with intraoperative fluoroscopy, potentially enhancing accuracy in stentgraft placement. These systems also promise to reduce the reliance on repeated fluoroscopic imaging, thereby lowering the overall radiation and contrast agent dose required during the procedure.

There are two studies available in the literature examining image fusion during TEVAR. Ahmad et al. [19] compared traditional 2D3D image fusion to a control group, while Barral et al. [20] examined the effects of traditional 2D3D image fusion compared to 3D3D image fusion in TEVAR cases. However, no existing data are available on the comparison of 2D3D and 3D3D image fusion with a control group in TEVAR.

With this paper, we aimed to compare the outcomes of 2D3D, 3D3D image fusion, and non-image fusion techniques for simple zone 2 and zone 3 TEVAR cases in terms of radiation exposure, contrast dose, and fusion and projection accuracy. For 2D3D fusion, we implemented a novel, stiff wire-based registration technique. This approach emphasizes the ongoing need for technological innovation and evidence-based refinement of procedural protocols to optimize outcomes for both patients and providers involved in endovascular repair.

## 2. Methods and Materials

### 2.1. Study Design

A single-center retrospective observational cohort study was conducted based on data gathered from patients who underwent TEVAR between 1 January 2016 and 31 May 2023 at our tertiary aortic referral center, using the search engine of our local informatic healthcare system. Inclusion criteria included any TEVAR procedure performed within the indicated time interval and proximal landing zones situated at either Z2 or Z3 locations. Patients who had previously undergone TEVAR were excluded. Follow-up was not applicable due to the study design. We adhered to the Strengthening the Reporting of Observational studies in Epidemiology reporting guideline.

Three cohorts were created based on the image fusion technique: TEVAR with 2D3D image fusion using stiff wire (2D3D-IF cohort), TEVAR with 3D3D image fusion (3D3D-IF cohort), and TEVAR with no image fusion (NIF cohort). The dose area product (DAP), number of pre-deployment angiographies (surrogate for contrast dose), and projection accuracy were measured and compared across all three groups, while image fusion accuracy was compared between the 2D3D IF and 3D3D IF groups.

### 2.2. Ethics

The study protocol was approved by the local Institutional Review Board (No. PRO00033063). The study was conducted in accordance with the principles of the Declaration of Helsinki.

### 2.3. Outcomes

The dose area product (DAP) and contrast dose were defined as primary outcomes. Projection accuracy and image fusion accuracy were considered secondary outcomes. In addition, in a subgroup analysis, Z3 TEVAR data were interrogated separately, free of the potential confounding effect of Z2 debranching.

Since the cases varied from simple to complex TEVAR, the decision was made to isolate the proximal stent graft implantation step to ensure comparability of the collected data across the cohorts.

We isolated the endograft implantation step to the proximal landing zone and included only those DSAs which were acquired during the pre-deployment phase. The end of the pre-deployment phase was marked by the angiography of the deployed stent graft. The corresponding DAPs were collected, and the DAP associated with fusion was added in each applicable case.

The number of pre-deployment angiographies was used as a surrogate for contrast dose. They were determined by identifying those DSAs which were acquired during the pre-deployment phase of TEVAR and were collected from syngo Dynamics (Siemens Healthineers, Malvern, PA, USA).

**Figure 1:**
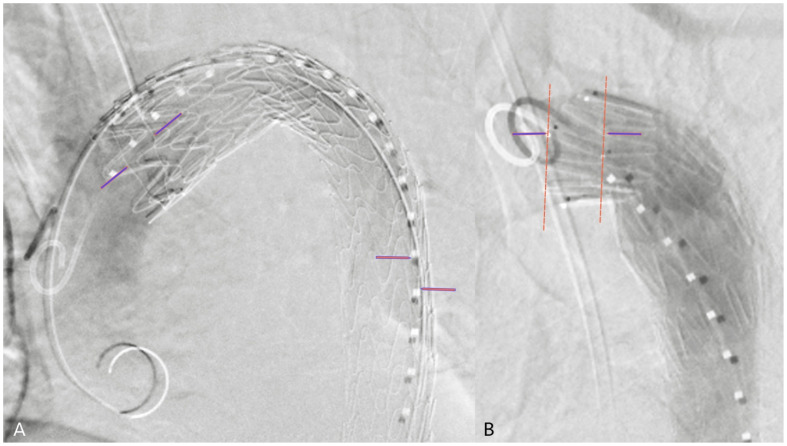
Projection accuracy was calculated based on the immediate DSA following stent graft deployment, by dividing proximal stent graft parallax with the correction value. In case of Gore devices, the proximal ring was used to calculate the parallax ((**A**), blue arrows). In those instances, where the ring was missing (non-Gore devices), two struts below each other were connected ((**B**), orange dotted lines) on the corresponding “anterior-posterior” sides of the graft and the distance was measured from the anterior strut to the posterior strut ((**B**), between blue arrows). The correction value was obtained by measuring the distance between the inner sides of markers ((**A**), orange arrow) on the sizing catheter, where the gap is the biggest between them.

The projection accuracy was defined as the ratio of the parallax (Figure 1A,B) observed on the proximal ring of the stent graft (or the parallax between the struts) and the distance between the sizing catheter markers (Figure 1A).

**Figure 2:**
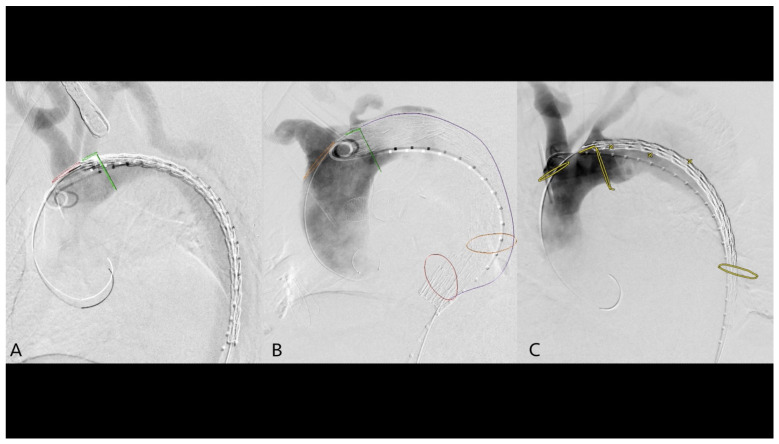
Three categories of image fusion accuracy: 0—perfect alignment of the annotated origin and actual origin—verified with DSA (**A**), 1—less than 50% misalignment (**B**), 2—misalignment above 50% (**C**). Abbreviation: DSA-digital subtraction angiography.

The image fusion accuracy was defined based on the misalignment of the annotation and the most distal supraaortic branch and its origin on DSA (Figure 2). Information on image fusion accuracy could not be obtained from 8 patients.

### 2.4. 2D3D Fusion Technique

**Figure 3:**
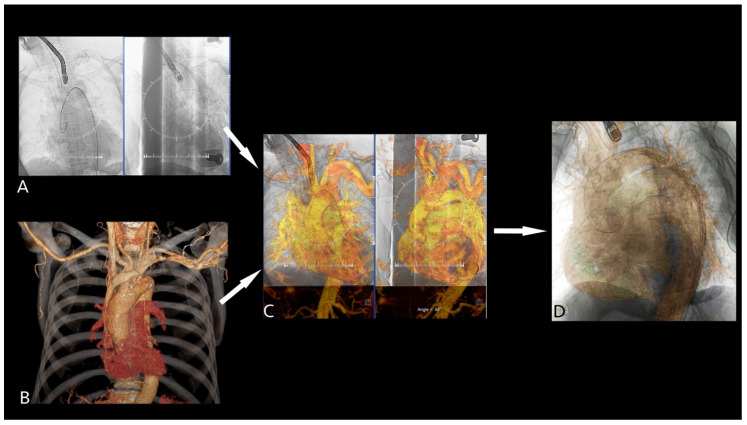
Following stiff wire introduction to the aortic arch, fluoroscopy was acquired in anterior and LAO 60 view (**A**). The preoperative CTA (**B**) was used for fusion (**C**). The anatomically relevant structures were annotated, and the mask was overlayed on the live fluoroscopy image (**D**). Abbreviations: CTA–computed tomography, LAO–left anterior oblique.

As an integral part of the preoperative planning, each patient underwent triphasic chest or chest-abdominal CTA imaging protocol. Based on the preoperative CTA TEVAR was indicated in each case as the optimal treatment strategy. For 2D3D image fusion, two fluoroscopic images (anteroposterior and left-anterior-oblique view) were acquired after the stiff wire (Lunderquist) was introduced to the aortic arch (Figure 3A)—a step that is routinely performed during TEVAR procedures. The stiff wire in the aortic arch was manually aligned with the aorta on the 3D preoperative CTA images (Figure 3B,C). After image fusion, the anatomically relevant landmarks were electronically annotated in the CTA and the mask was overlaid on the live fluoroscopic image to guide the endograft deployment (Figure 3D).

### 2.5. 3D3D Fusion Technique

**Figure 4:**
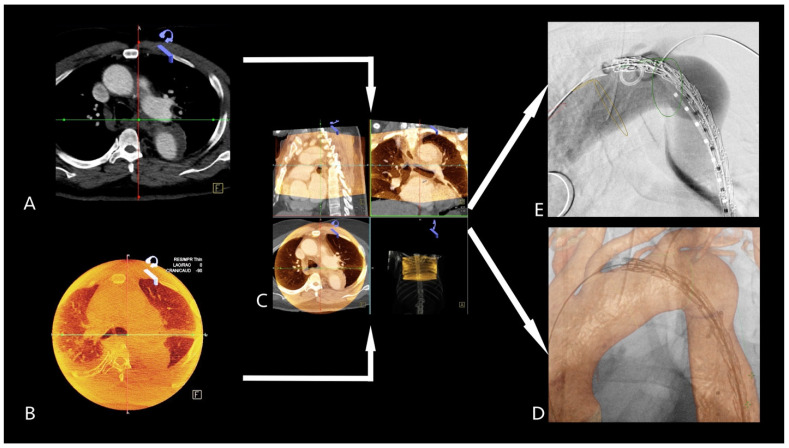
The preoperative CTA (**A**) and the intraoperative non-contrast CBCT (**B**) were used for fusion (**C**). The anatomically relevant structures were annotated, and the mask was overlayed on the live fluoroscopy image (**D**,**E**). Abbreviations: CTA–computed tomography, CBCT–cone-beam CT.

The intraoperative non-contrast cone-beam CT (CBCT) was acquired in a supine position. The thoracic vertebral bodies were used to align the preoperative CTA (Figure 4A) and the intraoperative CBCT (Figure 4B). After image fusion (Figure 4C), the anatomically relevant landmarks were electronically annotated in the CTA and the mask was overlaid on the live fluoroscopic image (Figure 4D,E) to guide endograft implantation.

### 2.6. Statistical Analysis

Categorical variables were assessed with Fisher’s exact test. Independent continuous data were analyzed with the Kruskal–Wallis test for comparison between the three cohorts. Post-hoc analysis was carried out using the Dunn test. Image fusion accuracy and DAP/DSA were assessed using the Mann–Whitney U test. Type I error was defined at 5%. Continuous data were presented in terms of median (interquartile range). Categorical data were presented in terms of frequency (percentage). The SPSS software (v28.0) was used for statistical analysis.

## 3. Results

**Figure 5:**
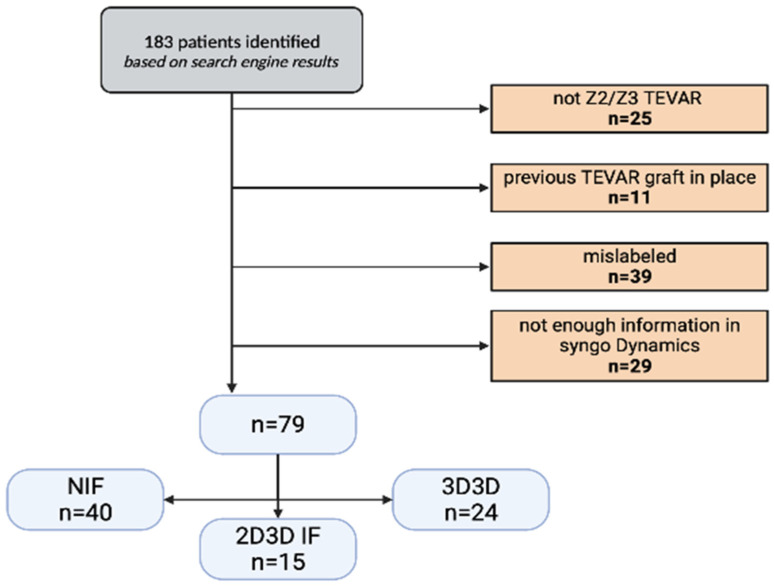
Flow chart, demonstrating the exclusion process and the final cohorts. Abbreviations: IF–image fusion, NIF–non-image fusion, TEVAR–thoracic endovascular aortic repair.

**Figure 6:**
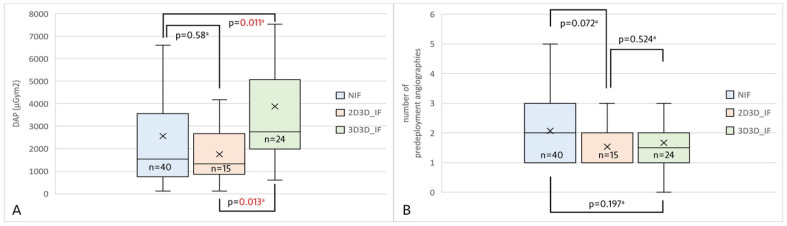
Box plot charts demonstrate the effect of the two image fusion techniques and non-image fusion during Z2 and Z3 TEVAR in terms of DAP (**A**), number of pre-deployment angiographies (**B**). Abbreviations: DAP–dose area product, IF–image fusion, NIF–non-image fusion, TEVAR–thoracic endovascular aortic repair. ^a^: post-hoc Dunn test.

A total of 183 patients were identified who had undergone TEVAR in the period concerned. Following the application of the inclusion and exclusion criteria (Figure 5), 79 patients were included. Three cohorts were created: NIF, 2D3D IF, and 3D3D IF. Demographic data, past medical history, and indication for TEVAR are displayed in Table 1. DAP was significantly lower in both the NIF [1542.75 µGym^2^ (751.72–3351.25 µGym^2^), *p* = 0.011] and 2D3D IF [1320.1 µGym^2^ (858.57–2572.07 µGym^2^), *p* = 0.013] groups compared to the 3D3D [2758.61 µGym^2^ (2074.73–4772.9 µGym^2^)] cohort (Figure 6A). The DAP in the 3D3D IF group was not offset by the cohort’s significantly lower BMI compared to the NIF cohort [ 24.89 kg/m^2^ (22.59–27.73 kg/m^2^) vs. 28.08 kg/m^2^ (25.49–32.92 kg/m^2^), *p* = 0.021]. In addition, a trend was observed toward a lower contrast dose in the 2D3D IF group compared to the NIF group [1 (1–2) vs. 2 (1–3), *p* = 0.073] (Figure 6B). Furthermore, a trend was shown toward more optimal projection accuracy in the 2D3D IF and 3D3D IF groups compared to the NIF group. The correction value used for projection accuracy calculation did not differ across the three groups (*p* = 0.803).

There was no statistically significant difference between 2D3D IF and 3D3D IF in terms of image fusion accuracy [1 (0.75–1.25), n = 13 vs. 2 (2–2), n = 18, *p* = 0.826].

### Z3 TEVAR Subgroup

**Figure 7:**
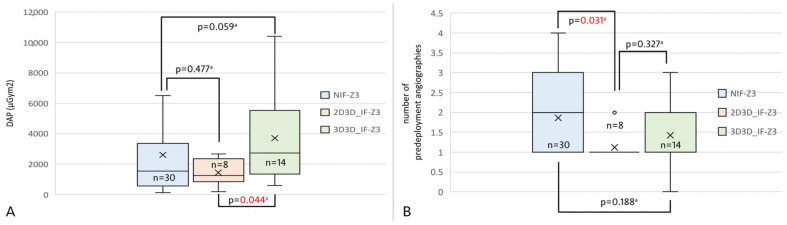
Box plot charts demonstrate the effect of the two image fusion techniques and non-image fusion in the Z3 TEVAR subgroup in terms of DAP (**A**), and the number of pre-deployment angiographies (**B**). Abbreviations: DAP–dose area product, IF–image fusion, NIF–non-image fusion, TEVAR–thoracic endovascular aortic repair. ^a^: post-hoc Dunn test.

A total of 30 patients underwent Z3 TEVAR in the NIF cohort, 8 and 14 patients in the 2D3D IF and 3D3D IF cohorts, respectively. DAP was significantly lower in the 2D3D IF group compared to the 3D3D IF group [(1270.84 µGym^2^ (860.56–2144.69 µGym^2^) vs. 2735.76 µGym^2^ (1583.86–5077.23 µGym^2^), *p* = 0.044], (Figure 7A). Additionally, 2D3D image fusion was associated with a significantly lower contrast dose compared to NIF [1 (1–1) vs. 2 (1–3), *p* = 0.031], (Figure 7B). Furthermore, the C-arm angulation was significantly more accurate in the 2D3D IF and 3D3D IF groups in comparison to NIF [0.4 (0.19–0.73) vs. 0.89 (0.68–1.33), *p* = 0.021] and [0.48 (0.16–0.73) vs. 0.89 (0.68–1.33), *p* = 0.009]. The correction value used for projection accuracy calculation did not differ significantly among the subgroups (*p* = 0.722).

Finally, the image fusion accuracy was not influenced by the IF technique [2D3D IF, 0.5 (0–1) vs. 0 (0–1), *p* = 0.818].

The subgroup analysis of DAP among patients undergoing TEVAR with the new X-ray system demonstrated similar results to the entire study population, showing a significantly higher radiation dose in the 3D3D fusion group [NIF (n = 24) 1190.92 µGym^2^ (542.1–2329.4 µGym^2^) vs. 2D3D IF (n = 10) 1156.39 µGym^2^ (622.08–2237.79 µGym^2^) vs. 3D3D IF (n = 10) 2500.93 µGym^2^ (2200.28–6011.96 µGym^2^), *p* = 0.021].

## 4. Discussion

It seems that Z2-Z3 TEVARs with 2D3D IF are associated with significantly less DAP compared to the 3D3D IF technique, with no additional cost in terms of radiation exposure compared to NIF. While the Z3 TEVAR subgroup resembled the findings of the entire study population in terms of DAP, the contrast dose was lower in the 2D3D cohort in comparison to NIF. One of the advantages of the wire-based 2D3D IF technique is the possibility of obtaining information on the location of the aorta, which is used for registration with the preoperative CTA. In contrast, intraoperative CBCT (non-contrast) used for 3D3D IF provides information on the bony landmarks. Although in our experience aortic calcification proved to be a reliable fiducial marker for 3D3D IF, it is not present in every patient. While 3D3D IF could aid in achieving an accurate projection angle comparable to 2D3D IF, it comes at the cost of significantly higher radiation exposure compared to both 2D3D IF and NIF. Furthermore, it fails to contribute to a lower number of DSAs, and therefore to reduced contrast consumption. Moreover, DAP exposure with 2D3D IF seems to be on par with NIF.

Consequently, stiff wire-based 2D3D IF seems to be the optimal choice over 3D3D IF in simple Z2-Z3 TEVAR. As far as Z3 TEVAR is concerned, our data suggest 2D3D IF over NIF and 3D3D IF as the favorable technique.

In addition to these findings, the use of 2D3D IF provides a clear procedural advantage by reducing the overall complexity of image acquisition. Unlike the CBCT-based 3D3D IF approach, which requires longer setup and acquisition times, the stiff-wire technique in 2D3D IF integrates seamlessly into the procedural workflow, minimizing delays and potentially reducing operator fatigue. This is particularly important in high-volume centers where procedural efficiency can significantly impact patient throughput and resource allocation. Furthermore, the ability to achieve precise anatomical registration without reliance on aortic calcifications broadens the applicability of 2D3D IF to a more diverse patient population, including younger individuals or those without significant vascular calcifications.

From a safety perspective, the lower contrast agent utilization observed with 2D3D IF is especially beneficial for patients with impaired renal function, reducing the risk of contrast-induced nephropathy.

While stiff wire-based 2D3D IF seems to be an excellent option to improve TEVAR cases, widespread adoption may be hindered by the abruption of the case to perform intraoperative image fusion after stiff wire placement. However, the adoption of this extra step in the workflow could unlock the potential of this particular technique.

Future studies could explore further optimizations of the stiff- wire-based 2D3D IF technique, including the integration of advanced imaging modalities or machine learning algorithms to enhance registration precision and reduce operator dependency. Additionally, cost-effectiveness analyses and long-term follow-up studies assessing patient outcomes could provide deeper insights into the clinical and economic impact of adopting stiff wire-based 2D3D IF as a standard technique in TEVAR.

There are limited data available in the literature regarding intraoperative image fusion in TEVAR. Those discussing 2D3D IF utilized a traditional, not wire-based approach.

Ahmad et al. [21] compared 2D3D IF (not wire-based) and NIF in TEVAR patients. The analysis of the entire study population showed a significant difference in the contrast dose (median, 70 mL [IQR, 50–101 mL]) compared with controls (median, 104 mL [IQR, 69–168 mL]; *p* < 0.001). Although the analysis of our entire study population suggested a trend toward lower contrast dose, statistical significance was not reached.

Based on the data of Ahmad et al., 2D3D IF in Z3 TEVAR resulted in significantly a lower contrast dose compared to the control group (64 mL [IQR, 43–81 mL] vs. 98 mL [IQR, 60–180 mL], (*p* = *0*.003)). Our findings are in line with Ahmad et al.’s data, by suggesting a significantly lower contrast volume in the 2D3D IF group compared to the NIF cohort. This finding is probably driven by the better projection accuracy reached in the 2D3D IF group.

Barral et al. [20] reported a randomized comparison of 2D3D IF and 3D3D IF in TEVAR with 32 patients. They found a significantly lower radiation dose in the 3D3D IF group (50.5 ± 30.1 Gy cm^2^ for 3D/3D vs. 99.5 ± 79.1 Gy cm^2^ for 2D/3D; *p* = 0.03) and more accurate fusion. In addition, according to their findings, the contrast dose was significantly lower in the 3D3D IF cohort (50.6 ± 22.9 mL vs. 98.4 ± 47.9 mL; *p* = 0.002). In contrast, we found the exact opposite in terms of DAP and did not identify a significant difference in terms of contrast dose and image fusion accuracy. The reason behind the difference in our findings compared to those of Barrel et al. might be two-fold. First, Barral et al.’s image fusion process for 2D3D fusion did not include electronic annotation of the proximal landing zone and the origin of the supraaortic branches, only the vascular volume, which might have compromised obtaining an accurate projection angle for endograft implantation. Second, they did not utilize stiff wire in the aortic arch for 2D3D IF. Stiff wire offers a favorable landmark as a fiducial marker for image registration in an anatomic region, where the ribs superimposing on the thoracic vertebral bodies render traditional vertebral-based registration rather challenging.

Both studies discussed above took the entire TEVAR procedure into account for the calculation of the contrast dose and DAP. In our opinion, only the step affected by image fusion should be examined and compared to obtain information on the true effect of the different intraoperative image fusion techniques in simple TEVAR in zones 2 and 3.

Limitations of our study include the single-center study design, the retrospective data collection, and the relatively low sample size, especially in the 2D3D IF cohort.

## 5. Conclusions

While stiff wire-based 2D3D IF requires advanced imaging equipment, vascular surgery units already utilizing traditional 2D3D IF might benefit from implementing our novel technique in their TEVAR workflow, by reducing DAP compared to 3D3D IF and providing better information on anatomical landmarks, compared to no fusion at all.

## Figures and Tables

**Table 1:** Abbreviations: BMI–body mass index, CAD–coronary artery disease, CKD–chronic kidney disease, DM–diabetes mellitus, IF–image fusion, LA–left atrium, NIF–non-image fusion, TAAD–type A aortic dissection, TBAD–type B aortic dissection, TEVAR–thoracic endovascular aortic repair.

	NIF (n = 40)	IF (n = 39)		*p*-Value
		2D3D IF (n = 15)	3D3D IF (n = 24)	
Age (year)	67.5 (59.75–76)	64 (54.5–72)	72 (67–78)	0.134 ^a^
Male/Female	28/12	25/14		0.105 ^b^
		9/6	16/8	-
BMI (kg/m^2^)	28.08 (25.49–32.92)	27.83 (23.47–31.67)	24.89 (22.59–27.73)	0.069 ^a^
NIF vs. 2D3D IF	28.08 (25.49–32.92)	27.83 (23.47–31.67)	-	0.547 ^c^
NIF vs. 3D3D IF	28.08 (25.49–32.92)	-	24.89 (22.59–27.73)	0.021 ^c^
2D3D IF vs. 3D3D IF	-	27.83 (23.47–31.67)	24.89 (22.59–27.73)	0.209 ^c^
Past medical history				
Atrial fibrillation	6 (15)	6 (15.38)		1.00 ^b^
		1 (6.7)	5 (20.8)	
Arterial hypertension	33 (82.5)	34 (87.18)		1.00 ^b^
		11 (73.33)	23 (92)	
CAD	4 (10)	3 (7.69)		1.00 ^b^
		0 (0)	3 (12)	
DM	7 (17.5)	5 (12.82)		1.00 ^b^
		3 (2)	2 (0.8)	
CKD	5 (12.5)	6 (15.38)		0.57 ^b^
		1 (6.67)	5 (2)	
Indication for TEVAR				
Aneurysm/pseudoaneurysm	9 (22.5)	13 (33.33)		0.32 ^b^
aortic dissection	26 (65)	20 (51.28)		0.258 ^b^
acute TBAD	15 (37.5)	13 (33.33)		
chronic TBAD	5 (12.5)	5 (12.82)		
residual TAAD	6 (15)	2 (5.13)		
Other	5 (12.5)	6 (15.38)		0.756 ^b^
PAU, IMH	5 (12.5)	5 (12.82)		
protective TEVAR before LA mass removal	0 (0)	1 (2.56)		
Emergency cases	19 (47.5)	10 (25.64)		0.062 ^b^

Categorical data are presented in terms of frequency (percentage). Continuous data are presented in terms of median (interquartile range). ^a^: Kruskal–Wallis test; ^b^: Fisher’s exact test; ^c^: post-hoc Dunn test.

## Data Availability

Dataset available upon request from the authors.

## Abbreviations

ALARA	As low as reasonably achievable
BMI	Body mass index
CBCT	Cone-beam CT
DAP	Dose area product
IF group	Image fusion group
NIF group	Non-image fusion group
OSR	Open surgical repair
TEVAR	Thoracic endovascular aortic repair
